# Physical and Mental Health Characteristics of Hospitalized COVID-19 Patients with and without Type 2 Diabetes Mellitus in Turkey

**DOI:** 10.3390/brainsci14040377

**Published:** 2024-04-13

**Authors:** Abdulbari Bener, Murat Atmaca, Abdulla O. A. A. Al-Hamaq, Antonio Ventriglio

**Affiliations:** 1 Department of Public Health, Medipol International School of Medicine, Istanbul Medipol University, Istanbul 34810, Turkey; abdulbari.bener@medipol.edu.tr; 2 Department of Evidence for Population Health Unit, School of Epidemiology and Health Sciences, The University of Manchester, Manchester M13 9PR, UK; 3 Department of Biostatistics & Medical Informatics, Cerrahpaşa Faculty of Medicine, Istanbul University-Cerrahpaşa, Istanbul 34320, Turkey; 4 Department of Endocrinology, Medipol International School of Medicine, Istanbul Medipol University, Istanbul 34810, Turkey; drmuratatmaca@hotmail.com; 5 Qatar Diabetic Association and Qatar Foundation for Research, Doha P.O. Box 752, Qatar; aalhamaq@qf.org.qa; 6 Department of Clinical & Experimental Medicine, University of Foggia, 71122 Foggia, Italy

**Keywords:** COVID-19, mental health, sleeping disorders, lifestyle, diabetes

## Abstract

The aim of this study was to assess the rates of depression, anxiety, and stress and quality of sleeping among COVID-19 patients with and without type 2 diabetes mellitus (T2DM). A case and control design has been employed, involving patients affected by COVID-19 infection (884 with T2DM vs. 884 controls without T2DM) and hospitalized in Istanbul (Turkey) from January to December 2021. A multivariate stepwise regression approach was used to test the associations between sociodemographic, metabolic, serum markers, mental health scores, and T2DM/COVID-19 patients’ clinical presentation. A statistically significant difference between T2DM and non-T2DM was found with respect to age, gender, BMI (body mass index), smoking, physical exercise, and physical comorbidities as well as levels of depression, anxiety, stress, and sleeping disorders (0.0003 ≤ all *p* = 0.025). With regard to serum biomarkers, vitamin D and ferritin were identified as useful parameters of reduction of glycated hemoglobin as well as COVID-19 infection among T2DM patients. This study detected that 25% of patients with COVID-19 and T2DM experienced mental distress, with sleeping disturbances and lifestyle changes markedly impacting their clinical outcome alongside metabolic and serum parameters.

## 1. Introduction

The COVID-19 pandemic has significantly impacted public health worldwide, and the lockdown has led to restrictions on the activities of daily life. Type 2 diabetes mellitus (T2DM) has been noted among the significant medical comorbidities reported by patients suffering from severe COVID-19 infection [1,2,3,4,5,6,7]. In fact, there have been several studies exploring the impact of COVID-19 infection on clinical outcomes of T2DM and how diabetes may increase the risk of severe respiratory syndromes in patients with coronavirus-related illness [1,2,3,4,5,6,7,8]. In fact, patients affected by diabetes appear to be at an increased risk of severe illness and complications if they contract COVID-19. This is mostly due to the fact that diabetes can weaken the immune system and reduces defenses against the infection [1,2,3,4,5,6,7,8]. Additionally, there is evidence that the expression and activity of ACE2 (angiotensin-converting enzyme 2) may be increased in certain tissues of people with diabetes, leading to higher susceptibility to COVID-19 infection and higher severity of disease [1,2,3,4,5,6,7,8]. In fact, it is well known that the interaction between the virus spike protein and the ACE2 receptors is crucial for the entry of the virus into the host cells [5,6].

In addition, it has been reported that the COVID-19 infection negatively affects carbohydrate metabolism, patients’ daily lifestyle, and dietary habits, leading to additional mental distress, lack of sleep, and reduced physical activity in T2DM patients [9,10,11,12,13,14,15]. Thus, illness, stress, and certain medications used to treat COVID-19 (e.g., corticosteroids) may all influence blood sugar levels, leading to difficult management of the insulin therapy or oral antidiabetic medications [9,10,11,12,13,14,15].

It has been also found that the clinical condition T2DM is highly associated with depression, anxiety, and stress symptoms over the course of illness. This comorbidity is due to the psychological adjustment of patients in the long term and some indirect consequences of metabolic dysfunctions over time (e.g., the impact of diabetes on the central nervous system) [16,17,18,19,20,21]. According to recent evidence, 33% of patients with T2DM report depressive symptoms, 11.2–26.3% report anxiety, and 39% report sleep disorders (also due to their obesity or obstructive sleep apnea) [16,17,18,19,20,21]. In addition, the lockdown restrictions during the first waves of the COVID-19 pandemic led to relevant changes in sleeping, eating, and physical exercise habits [15,16,17,18,19,20,21,22,23], secondarily increasing psychological loneliness, insomnia, depression, anxiety, and stress symptoms [24] and leading to subjective lower life satisfaction in the general population [16,17,18]. Regarding the impact of comorbid COVID-19 with T2DM on patients’ mental health, some evidence has suggested that 43% of them reported significant levels of depression and psychological distress; moreover, 77.5% of comorbid acute COVID-19 patients reported moderate/severe sleep disorders [25].

This study aimed to determine the rates of depression, anxiety, stress, and sleep disorders among COVID-19 patients with T2DM vs. without T2DM alongside their physical characteristics and comorbidities, including biochemical parameters, in order to evaluate the differences in terms of mental and physical health in patients with the comorbid infection (COVID-19 with T2DM), who are recognized as a subgroup vulnerable to severe effects of coronavirus-related illness. We also explored the role of physical and mental health characteristics in the clinical characterization of comorbidity between COVID-19 infection and T2DM using a multiple linear stepwise regression method.

## 2. Subjects and Methods

### 2.1. Study Population and Design

This study was based on a case–control design and conducted in the urban and rural hospitals of Istanbul, Turkey. In particular, patients affected by COVID-19 infection and diagnosed with T2DM (with a history of illness at least of 3 years, as suggested by the literature) [15] were compared to COVID-19 patients without T2DM, who were included as controls. Thus, all subjects hospitalized for COVID-19 infection from January to December 2021 were consecutively enrolled and grouped into T2DM patients and controls and tested to assess their physical and mental health (see Section 2.3) characteristics. All variables were measured or described upon patient intake (hospital admission), and the study design did not include any follow-up assessment. Exclusion criteria included serious co-morbidities such as unstable angina, any terminal cancer, severe hepatic/renal disease, elderly patients with alertness problems, and newly diagnosed T2DM. All patients reported being vaccinated for COVID-19 and being covered by their last dose of the vaccine (a second or third dose).

### 2.2. Sample Size Calculation

The sample size was based on the expected proportion in controls (*p* = 0.01), assumed odds ratio (OR = 4), confidence interval (CI = 0.99) and power = 0.80. The final sample was composed of 884 T2DM subjects vs. 884 controls (without T2DM), who were males and females aged 25–75 years old and all affected by COVID-19 infection. Recruitment was performed during the second peak of the COVID-19 pandemic from January to December 2021. Patients were also grouped in age categories (<45 years old; 45–54; 55–64; ≥65) according to evidence from the international literature suggesting that both COVID-19 infection and diabetes may show age-related differences in their characteristics and outcomes [26,27].

### 2.3. Methods and Measurements

This study considered patients’ sociodemographic variables, clinical characteristics, measurements concerning T2DM, other comorbidities than COVID-19 infection, and clinical biochemistry and clinical microbiology tests (which are used in COVID-19 diagnosis). Additionally, influenza polymerase Rt-PCR (reverse transcription polymerase chain reaction) testing was performed and recorded by the Turkish Ministry of Health (not shown in the manuscript). The rate of metabolic syndrome (MetS) was identified by assessing parameters stipulated in the National Cholesterol Education Program-Adult Treatment Panel III (NCEP-ATP III) and International Diabetic Federation (IDF) criteria [8]: (i) high blood pressure; (ii) waist circumference; (iii) hypertriglyceridemia; (iv) low HDL-C (high-density lipoprotein cholesterol) and (v) hyperglycemia.

A set of radiological investigations were considered, including radiographs (CXRs), magnetic resonance imaging (MRI), and computed tomography (CT), in patients with suspected COVID-19-related pneumonia. Radiological assessments were suggested by COVID-19, which was confirmed through real-time reverse transcription polymerase chain reaction (RT-PCR) [19]. These findings were not included in the report, since it did not have a radiological focus.

The psychometric assessment and the investigation of sleep quality were performed by employing the following rating scales, all standardized and validated in Turkey.

#### 2.3.1. The Pittsburgh Sleep Quality Index (PSQI)

The Pittsburgh Sleep Quality Index (PSQI) is a widely used self-reported questionnaire designed to assess the quality of sleep over a one-month time interval. It was developed by researchers at the University of Pittsburgh’s Sleep Disorders Center [23]. The PSQI consists of 19 items that generate 7 component scores, each representing a different aspect of sleep: subjective sleep quality; sleep latency (the amount of time it takes to fall asleep); sleep duration; habitual sleep efficiency (the percentage of time spent asleep while in bed); sleep disturbances (such as waking up during the night or having trouble breathing); use of sleeping medication; and daytime dysfunction (how sleep problems affect daily functioning). In this study, the scale was employed to assess any sleep disturbance among patients who were then divided into three groups: “good sleepers” with a PSQI score of ≤5; “average sleepers” with a PSQI score of 6–8; and “poor sleepers” with a PSQI of ≥9 [23,24].

#### 2.3.2. The Depression Anxiety Stress Scale (DASS-21)

The DASS-21 is a self-report questionnaire commonly used to assess the severity of symptoms related to depression, anxiety, and stress. It is a shorter version of the original DASS, which contains 42 items. The DASS-21 was developed by Lovibond and Lovibond in 1995 [24]. Each subscale consists of seven items, and respondents rate the extent to which they have experienced each symptom over the past week on a 4-point Likert scale ranging from 0 (did not apply to me at all) to 3 (applied to me very much, or most of the time). Total scores for each subscale can be calculated by summing the scores of the individual items, with higher scores indicating greater severity of symptoms. Additionally, there are established cutoff scores for each subscale to classify individuals into categories of normal, mild, moderate, severe, or extremely severe levels of depression, anxiety, and stress.

### 2.4. Ethical Approvals

Ethics Committee Approval was obtained from the Istanbul Medipol University Institutional Review Board (IRB# 10840098-604.01.01-E.14180).

### 2.5. Statistical Analysis

Statistical analyses employed commercial microcomputer programs (Statview 5, SAS Corp., Cary, NC, USA; Stata 18, Stata Corp., College Station, TX, USA). Statistical analysis was based on Student’s *t*-test in order to determine the significance of differences between mean values. The chi-square test was also used to test significance between two or more categorical groups. A multivariate stepwise regression analysis method was used, after adjusting for their age and gender, to test factors specifically associated with the clinical presentation of comorbid patients with T2DM. All analyses were two-sided, with a *p* value ≤ 0.05 considered statistically significant.

## 3. Results

Table 1 shows the comparison of sociodemographic characteristics between COVID-19 patients with T2DM and without T2DM. Significant differences between T2DM vs. control subjects were observed (*p* < 0.05) with respect to age, gender, BMI (body mass index), cigarette smoking, nargile/shisha smoking, and physical exercise. In particular, hospitalization rates for COVID-19 were higher among patients younger than 54 years old with T2DM but higher among patients older than 54 years in the control group. Females were more represented among T2DM patients and men among controls. Obesity was more represented among controls, whereas overweight was more represented among T2DM patients. Patients with T2DM also reported more smoking and nargile use as well as lower rates of daily physical activity.

Additionally, there was a statistically significant (*p* < 0.05) difference, as expected, between T2DM patients and control subjects regarding the rate of metabolic syndrome (ATP III and IDF), thyroid issues, chronic obstructive pulmonary disease (COPD), concurrent infections, history of stroke, coronary heart failure (CHF), malignancy, hypertension, and cardiovascular disease (Table 2).

**Figure 1:**
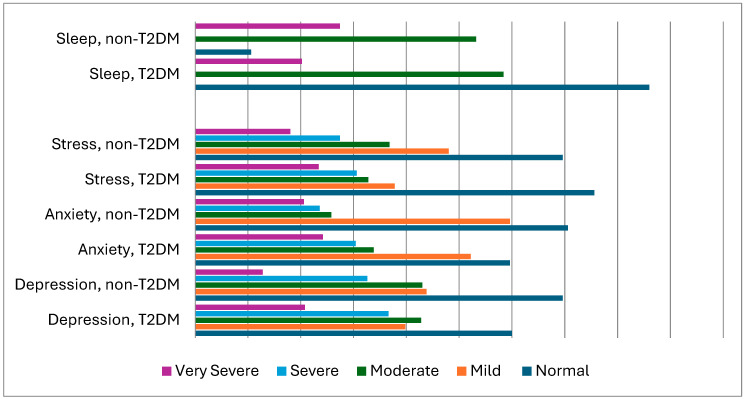
Assessment of mental health and sleeping quality among COVID-19 patients with T2DM (Type 2 Diabetes Mellitus) vs. without T2DM (controls) (*n* = 1768).

Table 3 and Figure 1 report the rates of concurrent depression, anxiety, and stress symptoms measured among T2DM vs. control subjects. The findings showed that the rates of depression (*p* = 0.009), anxiety (*p* = 0.003), stress (*p* = 0.025), and sleep disorders (*p* = 0.006) (as scored using the DASS-21 and Pittsburgh Sleep Quality Index (PSQI)), were all higher among T2DM patients vs. controls; vitamin D levels (*p* = 0.001) were lower among T2DM patients. Thus, 25% of T2DM/COVID-19 patients reported mental health issues as well as sleep disturbances.

Table 4 reports the additional significant differences found in the comparison between T2DM patients and control subjects, showing more abnormalities in the following serum biomarkers and vital parameters among T2DM patients, as expected: HbA1c (glycated hemoglobin), vitamin D, vitamin B12, calcium, HDL, fasting blood glucose, creatinine, triglyceride, uric acid, ferritin, TSH (thyrotropin), platelets, AST (aspartate transaminase), ALT (alanine transaminase), GGT (gamma-glutamyltransferase), and systolic and diastolic blood pressure levels. We considered these biochemical parameters in order to test their role, alongside other variables, in the clinical presentation of patients with comorbid COVID-19 infection in the following multivariate stepwise regression analysis.

Table 5 presents the findings of the analysis, which aimed to detect the role of specific factors in the clinical presentation of patients with comorbid COVID-19 and T2DM. Factors significantly associated were a lower number of red blood cells (*p* < 0.001), lower serum levels of vitamin D (*p* < 0.001), higher levels of HbA1c (*p* < 0.001), higher levels of creatinine (*p* < 0.001), higher levels of uric acid (*p*< 0.001), more smoking (*p*< 0.001), lower levels of vitamin B12 (*p*= 0.002), less physical activity (*p* < 0.011), lower PSQI sleep quality (*p* = 0.013), and higher incidence of metabolic syndrome (IDF) (*p* = 0.016). These factors were considered specific factors characterizing general health (serum biomarkers, metabolic syndrome, less physical activity and more smoking) and mental health (low quality of sleeping) in T2DM patients after adjusting for their age and gender.

## 4. Discussion

In this study, we assessed levels of anxiety, depression and sleep quality alongside clinical and biochemical variables among COVID-19 patients with T2DM vs. without T2DM.

Regarding the age of patients, it is of note that the number of hospitalizations for COVID-19 was higher in the group aged < 54 years old affected by T2DM (58.3%) than in non-T2DM patients (36.8%). This may confirm evidence [26,27] that the severity of COVID-19 infection may be higher in patients with comorbid diabetes, leading to higher hospitalizations even among young adults.

We found that the comorbid T2DM in COVID-19 patients was associated with lower quality of sleeping and higher levels of stress, depression, and anxiety. In particular, the alteration of sleep patterns, among other metabolic and lifestyle variables, was a specific factor associated with the clinical presentation of patients with COVID-19 and T2DM (Table 5). It is well known that psychological stress, lifestyle factors (including the quality of sleeping), and affective disorders all are associated with the changes in body weight, body mass index, and glycidic metabolism in the general population [2,3,4,5,6,7,8]. Thus, it may not be surprising that in this study, the proportion of COVID-19 subjects with T2DM reporting lower scores for mental health was higher than in other studies [1,2,3,4,12,28]. It has been also discussed that the lower metabolic control observed among COVID-19/T2DM patients may have been impacted by social distancing and stay-at-home policies, which led to unfavorable lifestyle changes and reduced physical activity, meaning worse glycemic balance [1,8,9,10,11,12,13,14]. Our results are in line with previous studies reporting that T2DM increased the risk of poorer outcomes from COVID-19 infection more than other risk factors [22]. Accordingly, evidence from other studies about COVID-19-related hospitalizations shows that the majority of patients admitted because of COVID-19 infection were older and reported co-morbid metabolic conditions including diabetes [2,4,21].

Specifically, alongside the metabolic and serum biomarkers reported in Table 5, some lifestyle factors, such as less physical activity and more smoking, were identified as associated with the clinical presentation of comorbid COVID-19 patients. Thus, among mental health factors assessed, poor sleep quality played a key role in the clinical characterization of those patients. This evidence is in line with recent findings from Turkey reporting a significant level of sleep disorders, fatigue, and depressive–anxious symptoms in the general population during the post-pandemic phase in 2022 [28]. In fact, the authors concluded that unsatisfactory sleep quality seemed to affect physical and mental health functioning in the general population [29]. Sleeping abnormalities have been broadly described as affecting the general population during the pandemic’s lockdown phase (not only in Turkey), particularly among people affected by COVID-19 [2,4,21]. Moreover, overall physical activity reduced during the COVID-19 lockdown, specifically among female and older adults with T2DM [21]. This trend of worse sleeping patterns has been largely replicated in other studies from Turkey [22], and Brazil [30], which all reported that abnormalities in sleep quality impacted mental health among adults during the first peaks of the COVID-19 pandemic. More recently, a report from Australia has shown that COVID-19 lockdown restrictions negatively impacted the quality of life, behavioral risk factors (including poor sleeping), and healthcare utilization among patients affected by T2DM [31]. This evidence confirmed again that COVID-19 infection negatively impacts the quality of life and lifestyle of adults with T2DM as well as their general clinical outcome. Comparing COVID-19 patients with and without T2DM, we confirmed that the comorbid infections were characterized by higher levels of stress, fear, depression, and anxiety (from the DASS-21), and these results are in line with a wide body of evidence from the international literature regarding the mental health consequences of COVID-19 [16,17,18,19,20,21]. Specifically, the rate of depression, distress and sleep disorders in our group of patients affected by COVID-19 infection and T2DM was 25%, which is a bit lower than those found in similar studies reporting significant mental health issues in 43% of comorbid patients (with sleep disorders affecting 77% of the assessed patients) [25]. These differences may be due to the different methods of detection employed and different sampling criteria (e.g., more or less acutely ill patients with severe respiratory syndrome). However, the literature on the mental health of comorbid patients is poor, and it is likely that more research studies are needed to add evidence to the available findings. In our sample, smoking, as a relevant lifestyle factor, was significantly associated with the clinical presentation of comorbid T2DM patients as they reported a higher use of cigarettes and nargile. We might argue that hospitalizations for COVID-19 in these patients were also due to their greater use of tobacco and nargile having a negative impact on their respiratory performances.

Regarding the metabolic outcome among patients with comorbid T2DM, COVD-19 infection impacted their HbA1c, showing an increase of +28.2%, as already found in similar studies [4,32,33]. Comorbid physical conditions (Table 2) were detected among T2DM comorbid patients, as expected, and confirmed that diabetes is related to a number of metabolic, cardiovascular, infective, and respiratory consequences [15,27]. Additional findings from our study also underlined that the ferritin level of subjects affected by COVID-19 with T2DM was significantly higher than that of the control subjects, as described in previous studies [34,35,36]. Of note, higher ferritin levels were also associated with higher levels of HbA1c, supporting the suggestion that ferritin may be a marker of elevated HbA1c in T2DM [35,36,37]. Finally, vitamin D was identified as a useful parameter of reduction in glycated hemoglobin and COVID-19 infection among T2DM patients.

### 4.1. Study Strengths and Limitations

To the best of our knowledge, this is one of the largest studies specifically aiming to measure sleep quality, depression, and anxiety among COVID-19 patients affected vs. non-affected by diabetes mellitus and also reporting on their metabolic, serum, and lifestyle factors. Nonetheless, our study has several limitations. Firstly, the design of this study is not based on an ideal match between cases and controls, since the patients were consecutively enrolled among those hospitalized for COVID-19 infection from January to December 2021 in Istanbul. Second, the recruitment of hospitalized patients biased the data collection because they were acutely ill, and we could not measure the impact of COVID-19/T2DM comorbidity on moderately or mildly ill patients. Third, we did not consider the oral glucose tolerance test among measurements (OGTT) in COVID-19 patients. Fourth, we could have employed more specific psychiatric ratings for the assessment of patients’ mental health, even if the tools used were sensitive and agile for a large sample of acute patients. Fifth, other mental health domains were not explored, e.g., psychotic symptoms, manic symptoms, substance abuse, etc. Sixth, other major comorbid clinical conditions might have impacted the outcomes of severely ill patients in both the sub- samples. Finally, in the assessment of sleep quality, we did not consider the employment of sleep medications and did not perform a specific sub-analysis.

### 4.2. Highlights

- The COVID-19 pandemic has significantly disrupted the daily life of patients with diabetes and impacted their clinical outcomes.
- Significant changes in HbA1c levels were confirmed among T2DM patients affected by COVID-19 infection.
- Some 25% of COVID-19 patients with T2DM have experienced pervasive psychological and mental burdens during the COVID-19 pandemic and COVID-19 infection in Turkey.
- The majority of T2DM patients reported a lack of sleep and lifestyle changes during COVID-19 infection. Low quality of sleeping, less physical exercise, and more smoking were, alongside other metabolic and serum parameters, factors specifically associated with the clinical presentation of these comorbid patients.
- Vitamin D and ferritin have been identified as useful parameters of reduction in glycated hemoglobin and COVID-19 infection among T2DM patients.

## 5. Conclusions

This study showed that twenty-five percent of COVID-19 patients with T2DM reported mental distress, sleeping disturbances, and lifestyle changes in addition to their physical symptoms. In a multivariate stepwise regression analysis model, low sleep quality and smoking were, alongside other lifestyle and serum variables, factors associated with the clinical presentation of comorbid COVID-19 patients with T2DM. We believe this report may add evidence to the debate on comorbidity between COVID-19 infection and diabetes.

## Figures and Tables

**Table 1:** Comparisons of sociodemographic variables between COVID-19 patients with T2DM (type 2 diabetes mellitus) vs. without T2DM (controls) (*n* = 1768).

Variables		T2DM, *n* = 884	Controls, *n* = 884	*p*-Value Significance
		*n* (%)	*n* (%)	
Age groups in Years				
	<45	286 (32.4)	141 (16.0)	
	45–54	229 (25.9)	184 (20.8)	0.001
	55–64	192(21.7)	231 (26.1)	
	≥65	177 (20.0)	328 (37.1)	
Gender				
	Males	360 (40.7)	434 (49.1)	0.001
	Females	524 (59.3)	450 (50.9)	
BMI				
	Normal (<25 kg/m^2^)	231 (26.1)	243 (27.5)	
	Overweight (29–30 kg/m^2^)	426 (48.2)	367 (41.5)	0.011
	Obese (>30 kg/m^2^)	227 (25.7)	274 (31.0)	
Smoking cigarette				
	Yes	168 (19.0)	127 (14.4)	0.009
	No	716 (81.0)	757 (85.6)	
Nargile smoking				
	Yes	161 (18.9)	120 (13.2)	0.008
	No	723 (81.1)	764 (86.8)	
				
Physical activity 30 min/day				
	Yes	221 (25.0)	273 (30.9)	0.006
	No	663 (75.0)	611(69.1)	

Note: BMI = body mass index.

**Table 2:** Comparisons of clinical variables between COVID-19 patients with T2DM (type 2 diabetes mellitus) vs. without T2DM (controls) (*n* = 1768).

Co-Morbidities Variables		T2DM, *n* = 884	Controls, *n* = 884	*p*-Value Significance
		*n* (%)	*n* (%)	
Metabolic Syndrome (ATP III)				
	Yes	195 (22.1)	157 (17.8)	0.024
	No	689(77.9)	727 (82.2)	
Metabolic Syndrome (IDF)				
	Yes	220 (24.9)	167 (18.9)	0.002
	No	664 (75.1)	717 (81.1)	
Thyroid Issues				
	Yes	254 (28.7)	112 (12.7)	0.001
	No	630 (71.3)	772 (87.3)	
Chronic Obstructive Pulmonary Disease (COPD)				
	Yes	257 (29.1)	140 (15.8)	0.001
	No	627 (70.9)	744 (84.2)	
Infection				
	Yes	250 (28.3)	207 (23.4)	0.019
	No	634 (71.7)	677 (76.6)	
Stroke				
	Yes	170 (19.2)	87 (9.80)	0.001
	No	714 (80.8)	797 (90.2)	
Coronary Heart Failure				
	Yes	222 (25.1)	163 (18.4)	0.001
	No	662 (74.9)	721 (81.6)	
Malignancy				
	Yes	108 (12.2)	63 (7.10)	0.001
	No	776 (87.8)	821 (92.9)	
Hypertension				
	Yes	210 (23.8)	125 (14.1)	0.001
	No	674 (76.2)	759 (85.9)	
Cardiovascular disease				
	Yes	187 (21.2)	93 (10.5)	0.001
	No	697 (78.8)	791 (89.5)	

Note: ATP III = Adult Treatment Panel III; IDF = International Diabetic Federation.

**Table 3:** Assessment of mental health, sleeping disorders, and vitamin D deficiency among COVID-19 patients with T2DM (type 2 diabetes mellitus) vs. without T2DM (controls) (*n* = 1768).

Variables and Scores	T2DM, *n*= 884 Yes *n* (%)	Controls, *n* = 884 Yes *n* (%)	*p*-Value Significance
Depression			
Normal (0–9)	265 (30.0)	308 (34.3)	
Mild (10–13)	176 (19.9)	194 (21.9)	
Moderate (14–20)	189 (21.4)	181 (21.5)	0.009
Severe (21–27)	162 (18.3)	144 (16.3)	
Very severe > 28	92 (10.4)	57 (6.44)	
Anxiety			
Normal (0–7)	263 (29.8)	312 (35.3)	
Mild (8–9)	231 (26.1)	263 (29.8)	
Moderate (10–14)	149 (16.9)	114 (12.9)	0.003
Severe (15–19)	134 (15.2)	104 (11.8)	
Very severe > 20	107 (12.1)	91 (10.3)	
Stress			
Normal (0–14)	334 (37.8)	308 (34.8)	
Mild (15–18)	167 (18.9)	212 (24.0)	
Moderate (19–25)	145 (16.4)	163 (18.4)	0.025
Severe (26–33)	135 (15.3)	121 (13.7)	
Very severe > 34	103 (11.7)	80 (9.00)	
Pittsburgh Sleep Quality Index:			
Good (PSQI ≤ 5)	380 (43.0)	445 (50.3)	
Average (6 ≤ PSQI ≤ 8)	258 (29.2)	235 (26.6)	0.006
Poor (PSQI > 8)	246 (27.8)	204 (29.1)	
Vitamin D Levels			
Deficiency < 20 ng/mL	552 (62.4)	466 (52.7)	
Insufficiency 20–29 ng/mL	243 (27.5)	297 (33.6)	0.001
Sufficiency ≥ 30 ng/mL	89 (10.1)	121 (13.7)	

**Table 4:** Comparisons of biochemistry variables between COVID-19 patients with T2DM (type 2 diabetes mellitus) vs. without T2DM (controls) (*n* = 1768).

Variables	T2DM, *n* = 884 Mean ± SD	Controls, *n* = 884 Mean ± SD	*p*-Value Significance
Hemoglobin (g/dL)	13.20 ± 0.49	13.59 ± 1.06	0.006
HbA1c	7.46 ± 0.81	5.65 ± 0.035	0.001
Fasting blood glucose (mmol/L)	135.18 ± 76.19	120.05 ± 56.23	0.009
Vitamin D (mmol/L)	18.02 ± 6.60	20.87 ± 7.28	0.001
Vitamin B12 (mmol/L)	252.68 ± 128.0	271.97 ± 11.96	0.001
Calcium (mmol/L)	1.72 ± 0.44	1.90 ± 0.27	0.001
Urea (mg/dL)	26.14 ± 3.37	32.003 ± 4.31	0.001
Phosphor (mmol/L)	1.74 ± 0.41	3.51 ± 1.22	0.001
Creatinine (mmol/L)	77.51 ± 19.14	72.10 ± 18.98	0.001
Total cholesterol (mmol/L)	166.52 ± 47.26	167.49 ± 44.10	0.665
HDL (mmol/L)	1.23 ± 0.27	1.29 ± 0.31	0.001
LDL (mmol/L)	180.49 ± 76.10	188.43 ± 89.91	0.343
Triglyceride (mmol/L)	164.49 ± 87.42	146.73 ± 97.58	0.001
Uric acid (mmol/L)	5.87 ± 2.19	5.42 ± 1.65	0.003
Ferritin (ug/L)	78.06 ± 18.984	72.10 ± 18.98	0.001
Fe (ug/L)	57.83 ± 28.97	59.13 ± 30.99	0.362
TSH	2.54 ± 1.18	1.71 ± 1.03	0.001
Creatine kinase (ug/L)	37.15 ± 18.47	36.97 ± 17.16	0.844
Creatine kinase–myocardial band (ug/L)	13.37 ± 6.24	13.21 ± 6.06	0.601
Hematocrit (ug/L)	36.191 ± 5.93	36.06 ± 5.76	0.631
White blood cells (×10^3^/µL)	7591.1 ± 1511.8	7649.2 ± 1507.5	0.415
Red blood cells (×10^3^/µL)	4.37 ± 0.63	4.19 ± 0.48	0.001
Neutrophils (×10^3^/µL)	5.76 ± 3.04	5.66 ± 3.00	0.487
Lymphocytes (×10^3^/µL)	1.63 ± 0.87	1.50 ± 0.90	0.230
Platelets (×10^3^/µL)	239.35 ± 94.88	227.52 ± 829.7	0.012
Aspartate transaminase (U/L)	27.25 ± 15.29	24.67 ± 11.370	0.001
Alanine transaminase (U/L)	24.86 ± 11.96	20.10 ± 7.56	0.001
C-reactive protein (mg/L)	8.95 ± 3.318	7.23 ± 3.32	0.001
Procalcitonin (ug/L)	0.24 ± 0.10	0.24 ± 010	0.873
Gamma-glutamyltransferase (GGT)	25.81 ± 15.58	23.97 ± 10.96	0.004
Systolic blood pressure (mmHg)	132.26 ± 13.59	130.34 ± 9.75	0.001
Diastolic blood pressure (mmHg)	79.31 ± 9.12	78.29 ± 6.98	0.008

Note: HbA1c = glycated hemoglobin; HDL = high-density lipoprotein; LDL = low-density lipoprotein; TSH = thyrotropin.

**Table 5:** Factors associated with the clinical presentation of comorbid COVID-19 infection and T2DM using a multiple linear stepwise regression method.

Independent Variables	Regression Coefficient	Standard Error	Beta	*t*-Test	*p*-Value Significance
Red blood cells (×10^3^/µL)	−0.144	0.021	−0.165	−7.016	0.001
Vitamin D deficiency (mmol/L)	0.093	0.017	0.132	5.581	0.001
HbA1c	−0.355	0.062	−0.787	−5.725	0.001
Creatinine (µg/L)	0.187	0.039	0.387	4.769	0.001
Uric acid (mmol/L)	−0.160	0.037	−1.057	−4.299	0.001
Smoking (Yes)	0.075	0.018	0.056	4.232	0.001
Vitamin B12 deficiency (mmol/L)	0.098	0.031	0.094	3.118	0.002
Physical vigorous activity	−0.039	0.015	−0.035	−2.554	0.011
PSQI sleep quality	−0.092	0.037	−0.059	−2.474	0.013
Metabolic syndrome (IDF)	0.075	0.031	0.062	2.402	0.016

Note: HbA1c = glycated hemoglobin; PSQI = Pittsburgh Sleep Quality Index; IDF = International Diabetic Federation.

## Data Availability

The datasets used and/or analyzed during the current study are available from the corresponding author on reasonable request due to ethical reasons.
